# Impact of Various Sleeve Materials on Temperature Variations During Guided Endodontic Access Cavity Preparation Utilizing Finite‐Element Analysis

**DOI:** 10.1002/cre2.70260

**Published:** 2026-01-09

**Authors:** Anna Muryani, Wandi Prasetia, Dudi Aripin, Hendra Dian Adhita Dharsono, Zainul Ahmad Rajion, Satrio Wicaksono

**Affiliations:** ^1^ Faculty of Medicine Universitas Padjadjaran Bandung West Java Indonesia; ^2^ Department of Conservative Dentistry, Faculty of Dentistry Universitas Padjadjaran Bandung West Java Indonesia; ^3^ Department Oral Maxillofacial Surgery and Oral Diagnosis Kulliyyah of Dentistry International Islamic University Malaysia Kuantan Malaysia; ^4^ Faculty of Mechanical and Aerospace Engineering Institut Teknologi Bandung Bandung West Java Indonesia

**Keywords:** access cavity preparation, cobalt‐chromium, finite‐element analysis, Guided endodontics, sleeve materials, thermal control, titanium, zirconia

## Abstract

**Purpose:**

Guided endodontics allows precise access in challenging cases such as calcified canals; however, drilling can generate temperature increases that risk damaging periradicular tissues. This study aimed to evaluate the influence of different sleeve materials—zirconia, cobalt‐chromium (CoCr), and titanium—on temperature changes during guided endodontic access using finite‐element analysis (FEA).

**Materials and Methods:**

High‐resolution three‐dimensional (3D) models of a human central incisor and titanium sleeve were developed using micro‐computed tomography (micro‐CT), 3D Slicer, Meshmixer, and SolidWorks. FEA simulations were conducted in Abaqus under a 2° deviation and 700 rpm drilling, with thermal properties based on literature. Experimental validation employed thermocouples to determine the temperature changes under identical conditions.

**Results:**

Results showed that zirconia sleeves produced the lowest temperature elevation (< 10°C) with localized concentration, while CoCr and titanium allowed more even heat dissipation.

**Conclusion:**

Zirconia is an effective insulator due to its thermal conductivity properties. CoCr has emerged as a promising alternative to titanium, offering more favorable thermal and mechanical characteristics.

## 1 Introduction

Guided endodontics has evolved as a novel technology for precisely finding and treating hardened root canals. In static guided procedures, computer‐generated, three‐dimensional (3D)‐printed surgical templates guide the drill along a predetermined trajectory into the pulp chamber. Clinical studies demonstrate that guided access significantly enhances canal detection and conserves tooth structure: for instance, one in vitro study revealed that guided templates facilitated canal identification in 91.7% of teeth, compared to merely 41.7% with the traditional freehand technique, while excising approximately 9.8 mm³ of dentin versus 49.9 mm³ through freehand preparation (Bansal et al. 2025; Connert et al. 2022, 2019; Muryani et al. 2025). These findings suggest that guided endodontics enhances the predictability and minimally invasive nature of root canal therapy, generally irrespective of the operator's expertise.

A guide sleeve is a cylindrical insert within the surgical template that supports and directs the bur during guided implant osteotomy (Kulinkovych‐Levchuk et al. 2022; Kim et al. 2021; Ozan et al. 2022). Although guide sleeves were traditionally manufactured from metal, contemporary designs incorporate bio compatible materials such as cobalt‐chromium (CoCr) alloys,titanium (Ti) alloys, and high‐strength ceramics, including zirconia (Ozan et al. 2022, Noorul et al. 2021; Shruthi et al. 2024; Liu et al. 2018; Rajnics et al. 2024). Previous in vitro studies have demonstrated that sleeve material influences wear behavior during repeated drilling, with zirconia sleeves showing reduced material degradation compared with metal sleeves, which may contribute to the maintenance of guiding accuracy over time (Noorul et al. 2021; Shruthi et al. 2024; Chen et al. 2021; Adams et al. 2023; El Kholy et al. 2019). Differences in thermal behavior between ceramic and metal material shave been reported in the materials science literature, particularly in restorative applications; however, the relevance of these thermal properties to sleeve guided implant surgery has not been established (Benetti et al. 2013).

Thermal considerations are critical during guided access cavity preparation. Friction at the rotating bur–tooth interface generates heat, and the enclosing guide sleeve can impede coolant flow, causing heat buildup (Kulinkovych‐Levchuk et al. 2022; Kim et al. 2021; Liu et al. 2018; Lau et al. 2023; Stocchero et al. 2021; Saxena et al. 2024; Zhang et al. 2022; Çelik Köycü and İmirzalıoğlu 2017; Li et al. 2024). Unlike alveolar bone, dentin and enamel lack blood perfusion, so heat generated in the canal is rapidly conducted to the periodontal ligament and surrounding bone. In guided implant surgery, surgeons have noted that use of a surgical guide can elevate intraosseous temperatures; by analogy, guided endodontic drilling (with thinner, longer burs) may produce even higher local temperatures. This is concerning because temperatures above ~47°C can induce irreversible injury in bone and periodontal tissues, and even moderate hyperthermia (~42°C–43°C) can impair cellular function and healing (Rajnics et al. 2024; Zhang et al. 2022; Sauk et al. 1988). Thus, managing heat in guided endodontics is essential for safety. Sleeve material may influence heat generation, with metal sleeves potentially conducting heat away from the bur into the guide, whereas an insulating zirconia sleeve may concentrate heat. However, the effect of sleeve thermal conductivity on temperature distribution within the tooth has not yet been quantified (Rajnics et al. 2024; Adams et al. 2023; Zhang et al. 2022).

Excessive heat generated during dental procedures can damage periodontal tissues and alveolar bone, with temperature increases above 10°C reported to cause ankylosis, resorption, or even necrosis (Gokturk et al. 2015; Özkoçak et al. 2015). Traditional thermocouples provide accurate point‐specific temperature measurements but are invasive and limited to discrete sites, whereas infrared thermography and infrared thermometers offer non‐contact, real‐time assessment of surface heat distribution (Mc Cullagh et al. 2000; Panov and Krasteva 2024). Studies in endodontics have shown that infrared imaging often detects higher and more widespread temperature elevations than thermocouples during post space preparation and warm gutta‐percha obturation, indicating potential risks to periradicular tissues (Gokturk et al. 2015; Er and Aslan 2015; Lipski 2006). In implantology, infrared thermography has demonstrated that guide design and irrigation significantly influence cortical heating, while thermocouple studies confirm that sleeve‐guided drilling produces measurable temperature rises but generally within safe thresholds (Özkoçak et al. 2015; Panov and Krasteva 2024). Together, these findings highlight the complementary roles of infrared imaging and thermocouple methods in ensuring thermal safety, supporting the optimization of endodontic and implant protocols to prevent heat‐induced tissue injury (Mc Cullagh et al. 2000; Stănuși et al. 2023).

Dental procedures often involve rapid thermal fluctuations (e.g., hot/cold foods or thermal cycling) that drive heat into teeth and restorations, potentially damaging tissues or compromising the integrity of restorations (Çelik Köycü and İmirzalıoğlu 2017). Finite‐element analysis (FEA) is capable of predicting these heat‐flow and stress patterns in complex tooth‐restoration systems. Study Koycu, et. al (2017) used 3D FEA to show that temperature changes concentrate thermal stress at the cervical enamel and that restorative materials with higher thermal expansion (such as composite resin) produce larger stress magnitudes than ceramic or metal inlays (Çelik Köycü and İmirzalıoğlu 2017; Li et al. 2024). In a similar way, Li et al. (2024) found that hygroscopic expansion of a resin support die during thermal cycling created a stress concentration at the crown margin and reduced the fracture resistance of ceramic crowns (Li et al. 2024). These results underscore that material thermal properties (conductivity, heat capacity, expansion, etc.) strongly influence intra‐tooth temperature gradients and stress distributions under thermal loads. In guided endodontic access, a drill sleeve embedded in the surgical guide channels the bur into the canal, so the sleeve's thermal conductivity and expansion behavior will likewise affect heat transfer into the tooth and any induced stress. Modeling different sleeve materials with FEA can therefore help optimize guided‐access design—ensuring minimal temperature rise and stress in the pulp and surrounding dentin during cavity preparation.

Prior FEA studies have shown that material thermal properties strongly affect intra‐tooth temperature gradients and stress under thermal loading (Çelik Köycü and İmirzalıoğlu 2017; Chien et al. 2021, 2023). By analogy, the thermal conductivity and expansion behavior of a guide sleeve should similarly influence heat transfer during drilling. A detailed three‐dimensional finite element model of guided access preparation, incorporating sleeve geometry, material properties, and realistic drill‐generated heat, enabled parametric comparisons of alternative sleeve materials (CoCr and zirconia) against titanium under identical conditions. The simulated temperature rise can be monitored at critical sites (interface) to assess how each material dissipates or retains heat (Liu et al. 2018; Rajnics et al. 2024; Stocchero et al. 2021; Saxena et al. 2024; Zhang et al. 2022; Möhlhenrich et al. 2015; Gmbh 2021).

Despite the importance of thermal safety and sleeve durability, a gap exists in the literature: most guided endodontic studies focus on canal localization accuracy, with few examining collateral effects, such as temperature rise. The thermal impact of different guide materials remains unclear. It is plausible that a sleeve's thermal conductivity and frictional behavior will affect heat generation and transfer, but these hypotheses have not been validated in an endodontic context. Understanding these factors is crucial, as selecting an optimal sleeve material could minimize the risk of thermal injury to the tooth and supporting tissues, while also improving guide longevity by reducing wear. In response, the present study used FEA to fill this knowledge gap. FEA was chosen as the first step because it enables a preliminary, detailed evaluation of temperature distribution as well as stress patterns that cannot be directly observed in experiments. Using FEA also reduces the number of experiments and research costs by allowing systematic parametric comparisons before manufacturing physical prototypes. A 3D finite element model of guided access drilling was developed to conduct a parametric study comparing three sleeve materials (CoCr, Ti, and Zirconia) under identical drilling parameters. Experimental validation was performed only on a commercially available titanium sleeve for endodontic guide drilling, which served as the control model, as CoCr and Zirconia sleeves were not yet available for endodontic guide drilling. Therefore, the objective of this parametric study is to evaluate sleeve temperature distribution resulting from operator‐induced angular deviation during access preparation, with sleeve material isolated as the primary variable. The findings will provide insight into how material choice influences heat generation, thereby guiding the selection of sleeve materials that optimize thermal management while maintaining guidance accuracy in clinical applications.

## 2 Materials and Methods

This research was conducted in the following sequence: micro‐computed tomography (micro‐CT) scanning, 3D modeling, finite element modeling, and analysis of temperature rise and stress distribution.

### 2.1 Micro‐CT Scanning

**Figure 1:**
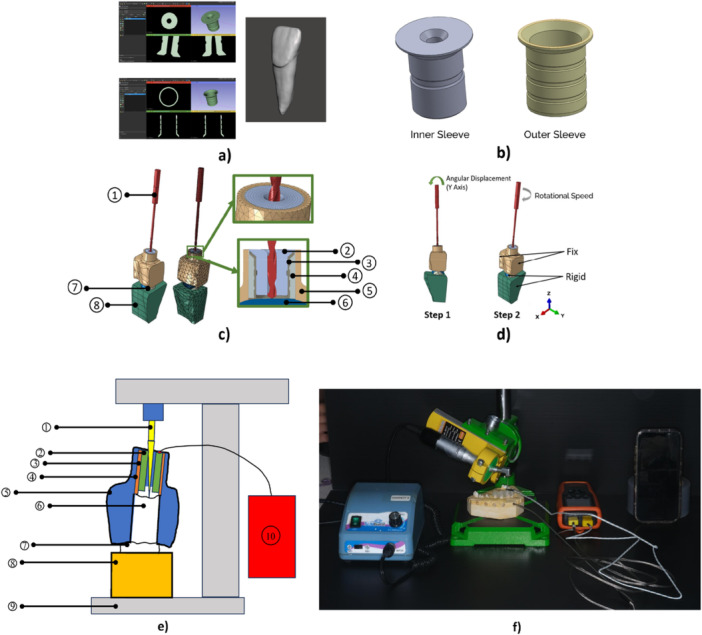
3D models: (a) sleeve and tooth micro‐CT scan results, (b) 3D model of sleeve, (c) finite‐element models with all components, and (d) loading and boundary conditions *†1: Drill, 2: Inner Sleeve, 3: Outer Sleeve, 4: Flowable Resin, 5: Template Resin, 6: Enamel, 7: Dentin, 8: Gypsum Base, 9: Drill Support, 10: Thermometer*, (e) Experimental Test Illustration, and (f) Experimental Test Configuration.

In this study, a commercially available titanium sleeve (control model) was purchased from the manufacturer. The sleeve was then scanned using a micro‐CT (computed tomography) imaging system (Micro‐CT Skyscan 1173, Skyscan Company, Antwerp, Belgium) with a pixel size of 5.36 µm. This scanning process was conducted to obtain the dimensional data of the sleeve. Additionally, a central incisor tooth was scanned to create a 3D model of the tooth. The scanning results for both the sleeve and the tooth are shown in Figure 1a.

The data from the scans were processed using 3D Slicer, Meshmixer, and SolidWorks software to generate accurate 3D models. The processing steps included reducing point cloud data, removing imperfections to ensure precise interpretation, and separating dentin from enamel in the tooth. Furthermore, dimensional measurements were conducted on the sleeve, with the results presented in Table 1.

**Table 1:** Dimensions of sleeve.

No.	Components	Dimensions (mm)
**Inner sleeve**		
1	Inner diameter	1.05
2	Outer diameter (head)	5.0
3	Outer diameter (body)	3.5
4	Height	5.0
**Outer sleeve**		
1	Inner diameter	3.5
2	Outer diameter (head)	5.0
3	Outer diameter (body)	4.0
4	Height	5.0

### 2.2 3D Modeling

The inner and outer sleeve models were created based on the dimensional measurement data. Several components, including a 1 mm diameter drill, flowable resin, template resin, and base, were generated using Meshmixer and SolidWorks to simulate the drilling process. The final 3D models are shown in Figure 1b,c.

### 2.3 Finite Element Modeling

Finite element (FE) models were developed from 3D geometries using the Abaqus software. Tetrahedral elements were used for most components, while the inner and outer sleeves were meshed with hexahedral elements to improve accuracy in critical regions. Three materials were investigated in this parametric study: titanium (control model), CoCr, and zirconia. Among the analyzed materials for the sleeve, titanium and CoCr are ductile, whereas zirconia is classified as a brittle ceramic.

The bone and base were modeled as rigid bodies to reduce computational time, as their distance from the active drilling zone resulted in minimal stress involvement. Similarly, dentin was modeled as a rigid body, while the drilling process interacted only with the enamel, for which no failure model was applied. The simulation primarily focused on the angular displacement of the drill bit relative to the sleeve. All other components (Sleeve, enamel, template resin, flowable resin, and drill) were defined as deformable to capture their mechanical behavior accurately under loading. The modulus of elasticity for tungsten carbide drill was 535 GPa and its tensile strength was 344 MPa (Zhao et al. 2019; Cardarelli 2008). Additionally, the material properties for sleeve models used in the study are presented in Tables 2 and 3.

**Table 2:** Material properties of sleeve.

No.	Material properties	Titanium	Zirconia	Cobalt‐chromium	Unit
1	Young's modulus (E)	102 (Elias et al. 2015)	206 (Dhital et al. 2020)	217 (Konieczny et al. 2020)	GPa
2	Poisson's ratio (v)	0.32 (Nagasawa et al. 2008)	0.3 (Dhital et al. 2020)	0.33 (Wang and Lim 2019)	
3	Yield strength	483 (Elias et al. 2015)	—	667 (Revilla‐León and Özcan 2017)	MPa
4	Tensile strength	550 (Elias et al. 2015)	750 (White et al. 2005)	1213 (Konieczny et al. 2020)	MPa
5	Compressive strength	—	2000 (Lagodzinska et al. (2023)	—	MPa
6	Specific heat	520 (Li et al. 2019)	466 (Dhital et al. 2020)	470 (Li et al. 2015)	J/Kg.K
7	Coefficient of thermal expansion	9 × 10^−6^ (Svanborg et al. 2024)	10 × 10^−6^ (Dhital et al. 2020)	14.3 × 10^−6^ (Konieczny et al. 2020)	/C
8	Thermal conductivity	22 (Li et al. 2019)	2.92 (Dhital et al. 2020)	22.77 (El‐Bediwi et al. 2009)	W/m.K
9	Density	4500 (Li et al. 2019)	6010 (Dhital et al. 2020)	8488 (Konieczny et al. 2020)	Kg/m3

**Table 3:** Coefficient of friction for sleeve materials.

No.	Components	Coefficient of friction
1	Carbide—titanium	0.43 (Abbas et al. 2023)
2	Carbide—cobalt‐chromium	0.36 (Wu et al. 2023)
3	Carbide—zirconia	0.46 (Bonny et al. 2009)

The drilling simulation was conducted by using Abaqus/Explicit (Temp‐Disp, Dynamics). The model was divided into Step 1—Angular Displacement and Step 2—Drill Rotation. In Step 1—Angular Displacement, the drill was subjected to an angular displacement of 2 degrees (Li et al. 2025) along the Y‐axis, as shown in Figure 1d. This angular displacement represents the deviation of the operator's hand during drilling. If the operator's hand fails to maintain the drill perfectly straight within the sleeve, the drill may come into contact with the inner sleeve wall. This contact creates additional pressure and friction forces during drilling, which can potentially increase the drill's temperature. In Step 2—Drill Rotation, the drill, positioned at the angulated displacement, was assigned a rotational speed of 700 rpm (73 rad/s). The template resin was restrained by rigidly fixing the flat surfaces in three directions. At the same time, the rigid bodies of dentin, bone, and base were also restrained at their reference point in three directions. The loading and boundary conditions are represented in Figure 1d.

A convergence test was performed to determine the optimal number of elements for the simulation. It was found that 83,134 elements were optimal for the drilling simulation, with a relative error of 6.6%. The convergence model utilized the following element sizes: the inner sleeve had a global element size of 0.2 mm and a local element size of 0.03 mm around the hole; the outer sleeve had a global element size of 0.1 mm and a local element size of 0.09 mm along the circumference; and the drill had a global element size of 0.8 mm with a refined local element size of 0.2 mm at the tip. Furthermore, almost all finite element models were linear elastic and limited to heat transfer conduction analysis, except that the titanium and CoCr sleeves were modeled as elastic‐plastic. All stress results are presented in Megapascals (MPa), equivalent to 1,000,000 Pascal in SI units, while temperature rise results are reported in degrees Celsius (°C), equivalent to 273 Kelvin.

### 2.4 Experimental Setup

An experimental study was conducted to validate the titanium sleeve as the control specimen for the FEA of the titanium sleeve. The titanium sleeve was modeled based on a commercially available product. In contrast, sleeves made of CoCr and Zirconia are not yet available. Therefore, the validation was performed exclusively on the titanium sleeve, while other materials were used in the FEA for this parametric study. The experimental setup comprised several components: a drill support, inner and outer titanium sleeves, a tooth, a resin template, a gypsum base for the tooth, and a thermocouple connected to a YET‐620/620L Two Channels High Accuracy Thermometer with a resolution of 0.01°C, which had been calibrated before the test. The outer sleeve was positioned on the resin template and bonded using flowable resin. The inner sleeve was then inserted into the outer sleeve. The tooth was embedded in the gypsum base. The drill that was inserted in the motor was gripped by the support. The illustration of the experiment is shown in Figure 1e,f.

The experiment was conducted at a rotational speed of 700 rpm (73 rad/s) with an additional angular displacement of 2 degrees Li et al. 2025. The temperature was measured using a YET‐620/620L thermometer equipped with a thermocouple, which was attached to the upper inner surface of the sleeve. Temperature measurements obtained from the experiment were compared with the simulation results to validate the finite element model. The experiment was conducted without irrigation to focus on the impact of sleeve material investigation. Additional investigations were carried out to compare the post‐drilling phenomena observed on the drill bit and sleeve with the corresponding simulation results. The drill bit was examined using Scanning Electron Microscopy (SEM, HITACHI SU3500, Japan) at 1000× magnification to verify drill damage. The sleeve surface was analyzed using Atomic Force Microscopy (AFM, AFM5300E, HITACHI High‐Tech, Singapore) at 2000× magnification to assess its surface condition. SEM and AFM results will be visually compared with the finite element results.

## 3 Results

A comparison between experimental and simulated results of the titanium sleeve revealed a temperature rise difference of only 9% after 1 s, with the experimental test yielding 0.63°C and the simulation 0.57°C. The simulation result was considered valid for the control model (Titanium Sleeve) because the error margin was below 10%. Therefore, the finite element models were deemed appropriate for the parametric study of other materials presented in this study.

**Figure 2:**
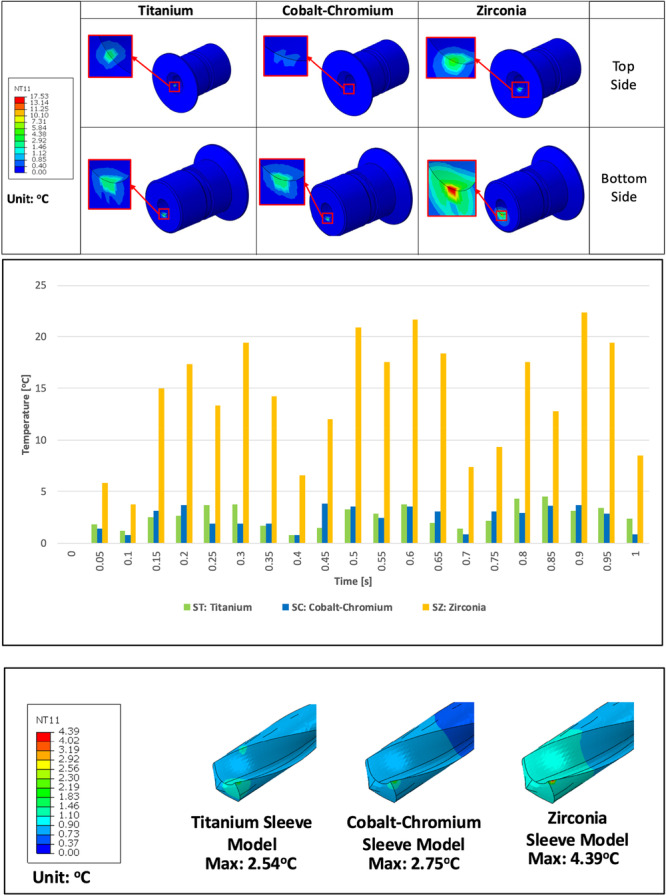
Temperature rise results with different sleeve materials in Step 2––Drill rotation. *†NT11: Nodal Temperature*.

A temperature rise analysis was conducted in this study. The results showed that the Zirconia Model (SZ) generated the highest temperature (with a maximum difference of up to 621%) on the inner sleeve, compared to the Titanium Model (ST) and CoCr Model (SC). ST and SC models exhibit similar temperature rises, despite the ST model showing higher temperatures (with a maximum difference of up to 64%). The rise in temperature of the inner sleeve during the 1‐second simulation is shown in Figure 2.

Moreover, the temperature rise on the drill was also analyzed. Based on the results in Figure 2, the drill in the SZ model exhibited the highest temperature rise, followed by the ST model and then the SC model. The drill in the SZ model showed a 73% difference compared to the drill in the ST model. On the other hand, the drill in the SC model experienced an 8% higher maximum temperature compared to the drill in the ST model.

**Figure 3:**
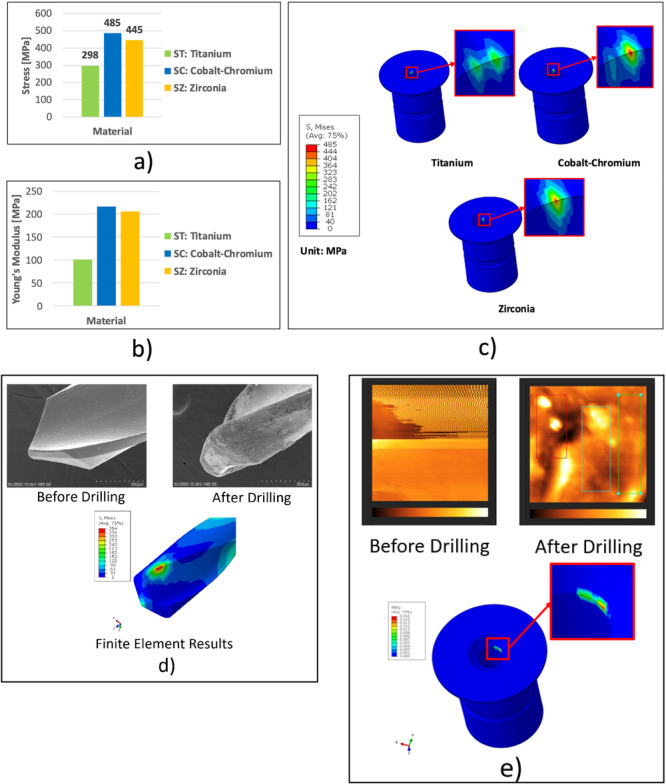
Stress results in Step 1—angular displacement: (a) stress value for different sleeve materials, (b) young′s modulus of materials, (c) stress distribution, (d) drill tip condition in experimental test and finite‐element analysis, and (e) surface condition of sleeve after drilling in experimental test and finite‐element analysis. *†S, Mises: von Mises Stress, PEEQ: Equivalent Plastic Strain Deformation*.

**Figure 4:**
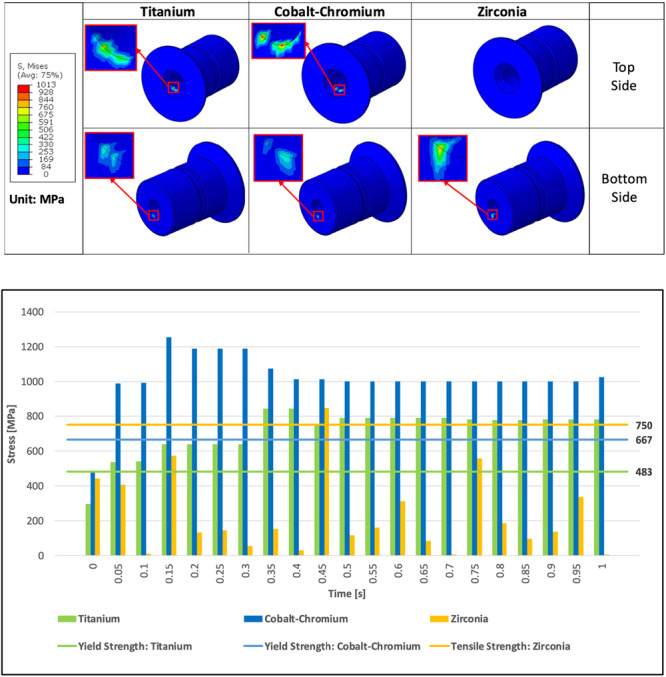
Stress results with different sleeve materials in Step 2—Drill Rotation. *†S, Mises: von Mises Stress*.

Additionally, an additional analysis was also conducted to verify the stress generated on the sleeve. In Step 1—Angular Displacement, the drill was angularly displaced by 2 degrees. This step generated additional pressure on the inner sleeve. Based on the results, all von Mises stress remained below the yield and tensile strengths of the materials. The Step 1––Angular Displacement stress results are shown in Figure 3a,c. The ST model experienced the least maximum von Mises stress among all configurations. The maximum von Mises stress in the CoCr model (SC) and the Zirconia model (SZ) was 63% and 49% higher, respectively, compared to the ST model. Moreover, the maximum von Mises stress in the SC model was 9% higher than in the SZ model. The von Mises stress results in Step 2—Drill Rotation were fluctuated due to the rotational movement of the drill combined with the additional pressure from the angular displacement. The results from 1 s of the drilling simulation indicated that the von Mises stress in all models did exceed the material's yield or tensile strengths, as shown in Figure 4.

Furthermore, SEM images (Figure 3d) revealed the presence of micro‐cracks and smearing on the tungsten carbide drill tip after the drilling process. These features are indicative of fatigue wear caused by high stress. On the other hand, finite element simulations predicted a peak stress of 364 MPa on the surface of the drill tip, which exceeds the tensile strength of the drill material (344 MPa). Finite element results indicated a potential local failure at the drill tip, consistent with the microstructural damage observed in the SEM result.

AFM analysis of the sleeve surface (Figure 3e) showed plastic deformation at the edge of the titanium sleeve hole where the drill made contact. The arithmetical mean height (Sa) decreased from 55.74 nm to 12.18 nm, and the slope (Δa) from 39.47° to 0.38°, indicating compression under mechanical stress. These findings suggest that high compressive forces and frictional heating softened the surface layer, causing plastic deformation. Experimental observations and finite element simulations both showed deformation at the sleeve edge, confirming good agreement between the two. SEM and AFM results confirmed that the phenomenon observed in the experiment and simulation is similar.

## 4 Discussion

The highest temperature rise in the endodontic guided sleeve with Zirconia (SZ) occurred due to zirconia's low specific heat, which causes the sleeve's temperature to change more easily compared to other materials. Additionally, zirconia's low thermal conductivity produced poor heat distribution within the sleeve, leading to a more localized temperature rise that is not effectively transferred to surrounding surfaces. The endodontic guided ST exhibited the second‐highest temperature rise in the study due to its high coefficient of friction with carbide. On the other hand, the endodontic guided sleeve with the CoCr (SC) generated the lowest temperature rise due to the low coefficient of friction between the carbide and CoCr, combined with CoCr good thermal conductivity, which effectively distributed heat to other surfaces.

Surgical guide sleeves made of Zirconia were found to sustain significantly less wear than CoCr surgical guide sleeves during surgical guided drilling Guz et al. Surgical guide sleeves madeHowever, zirconia's low thermal conductivity causes rapid heating, creating extremely steep temperature gradients (≈100°C in a zirconia vs. ≈30°C in a metal) (Benetti et al. 2013), suggesting that metal sleeves may dissipate heat more evenly during fast drilling. Drilling in molar regions has also been associated with increased sleeve wear and potentially greater heat generation (Guz et al.) highlighting that both sleeve material and drilling site strongly influence heat buildup, which is an important consideration since temperature increases of ~10°C at the root surface can damage bone (Kwon et al. 2013).

The temperature rise on the drill was primarily caused by frictional contact between the inner sleeve and the drill. The drill in the SZ model experienced the highest temperature rise among the models due to its higher coefficient of friction with carbide, followed by ST and SC. This heat was generated by the contact between the drill and the inner sleeve, compounded by the additional pressure from the angular displacement. During prolonged drilling, a drill with a high temperature rise may have the possibility to transfer more heat to the tooth during endodontic access preparation. On the other hand, guided endodontic access preparation has been shown to be highly accurate and successful (Moreno‐Rabié et al. 2020), enabling minimally invasive canal access. In one in vitro study, creating an initial access cavity prior to guided drilling, using a slower drill speed (800 rpm), and applying continuous cold irrigation each significantly reduced the temperature rise during guided drilling (Rajnics et al. 2024). Notably, sleeve design itself affects heat generation: a sleeveless guided system produced significantly lower temperature rise than a conventional sleeve‐guided system (Jin et al. 2025), suggesting that sleeve material and geometry are important factors in controlling thermal outcomes during guided endodontic access.

FEA studies have also demonstrated that the stress on NiTi files during canal preparation is primarily driven by insertion forces and canal curvature, with minimal additional stress resulting from continuous rotation (Chien et al. 2021, 2023; Roda‐Casanova et al. 2021). Chien et al. reported that von Mises stress sharply increases during the insertion phase, while Roda‐Casanova et al. demonstrated that a narrower canal radius significantly elevates stress and shortens fatigue life (Roda‐Casanova et al. 2021). These findings suggest that a guide sleeve, through its rigidity and contact area, may alter file stress distribution and frictional heat generation. Hard metal sleeves could restrict file motion and increase bending strains, leading to higher heat, whereas more flexible or lubricated sleeves might reduce contact loads. Because NiTi fatigue is temperature‐dependent, higher temperatures may accelerate phase transformations and reduce fatigue life (Chien et al. 2023; Roda‐Casanova et al. 2021). Consequently, sleeve thermal properties become clinically relevant. Metal sleeves may act as heat sinks, reducing temperature spikes, while insulating sleeves can retain heat at their interface. However, FEA outcomes are susceptible to boundary conditions, contact modeling, the mechanical stiffness, friction coefficient, and thermal conductivity of sleeve materials. All of these factors must be carefully considered when predicting heat production and dissipation (Chien et al. 2023).

Furthermore, due to its lowest Young's modulus among the evaluated materials, the inner ST experienced the lowest maximum stress. In contrast, the sleeve with the CoCr model (SC) experienced the highest stress, followed by the Zirconia model (SZ). This trend in sleeve stress corresponded closely with the Young's modulus of materials, where CoCr had the highest, followed by Zirconia and then Titanium, as illustrated in Figure 3b. With the 2‐degree angular displacement used to simulate the operator's hand movement without rotation, the stress on the sleeve and the drill did not reach their yield or tensile strengths. However, if the angular displacement were to increase, failure might occur in the drill or sleeve.

During the drilling process, stress levels on the sleeves increased, as shown in Figure 3. ST and SC models were designed with elastic‐plastic behavior among the materials tested. The SC model showed the highest stress, followed by the ST model and then the SZ model. This condition was attributed to its localized plastic deformation capacity, allowing it to absorb energy and conform to the drill's slight misalignment. The plasticity maintained near‐continuous contact with the rotating drill by dampening vibrations and minimizing abrupt separation. In contrast, the SZ model was designed as linear elastic and experienced lower but more fluctuating stresses due to repeated loss of contact. The primary cause of contact loss and bouncing in the SZ sleeve was its inability to dissipate energy. Under rotational speed of 700 rpm and slight tilt (two degrees), these materials stored mechanical energy during deformation and released it rapidly, resulting in elastic rebound.

In guided endodontic procedures, “periodontal tissues” refers to the supporting structures of the tooth, specifically the alveolar bone and periodontal ligament. Despite the high surgical accuracy provided by 3D‐template‐guided access, these tissues remain susceptible to collateral thermal damage from drill‐generated heat (Moreno‐Rabié et al. 2020). Consequently, clinicians utilize meticulous techniques, such as reduced drill speeds and coolant irrigation, along with careful selection of drill and guide materials, to minimize heat generation and protect these tissues (Rajnics et al. 2024; Moreno‐Rabié et al. 2020). Due to the limitations of this study, certain factors were not accounted for in the models. For future research, experimental tests of CoCr and zirconia will be conducted to analyze the different material effects on the sleeve during drilling. Additionally, parameters such as sleeve height and angular deviations at the guide‐positioning stage will be analyzed. At the same time, the scope of the study will be expanded to include temperature rise and stress distribution in the pulp chamber and root canal.

## 5 Conclusions

1. The choice of material plays a crucial role in distributing temperature and stress within the sleeve and the tooth during the pulp chamber access procedure. Selecting an appropriate material is important to ensure the sleeve can be used safely and effectively.
2. Zirconia is an effective insulator, as indicated by the higher localized temperatures observed in the zirconia sleeve, which could increase further during prolonged drilling. In addition, zirconia is a brittle material that may be more difficult to manufacture due to higher risk of fracture.
3. CoCr emerges as a potential alternative to titanium for guided endodontic sleeves, as it demonstrates better control over temperature compared to Titanium.

## Author Contributions

Anna Muryani contributed to conceptualization, methodology, supervision, data interpretation, and writing the original draft. Wandi Prasetia and Satrio Wicaksono contributed to methodology, finite element modeling, and formal analysis. Dudi Aripin and Hendra Dian Adhita Dharsono contributed to study design, validation, and manuscript review and editing. Zainul Ahmad Rajion contributed to clinical input, interpretation of results, and manuscript review and editing. All authors approved the final manuscript.

## Conflicts of Interest

The authors declare no conflicts of interest.

## Data Availability

The datasets generated and analyzed during the current study are available from the corresponding author on reasonable request; however, raw files cannot be shared publicly due to institutional limitations.
